# Cytomegalovirus Infection and Inflammation in Developing Brain

**DOI:** 10.3390/v13061078

**Published:** 2021-06-04

**Authors:** Fran Krstanović, William J. Britt, Stipan Jonjić, Ilija Brizić

**Affiliations:** 1 Center for Proteomics and Department of Histology and Embryology, Faculty of Medicine, University of Rijeka, 51000 Rijeka, Croatia; fran.krstanovic@medri.uniri.hr (F.K.); stipan.jonjic@medri.uniri.hr (S.J.); 2 Department of Pediatrics, University of Alabama at Birmingham, Birmingham, AL 35294, USA; WBritt@peds.uab.edu

**Keywords:** cytomegalovirus, congenital HCMV infection, mouse cytomegalovirus, inflammation, latency, central nervous system, CMV tropism, immune response

## Abstract

Human cytomegalovirus (HCMV) is a highly prevalent herpesvirus that can cause severe disease in immunocompromised individuals and immunologically immature fetuses and newborns. Most infected newborns are able to resolve the infection without developing sequelae. However, in severe cases, congenital HCMV infection can result in life-threatening pathologies and permanent damage of organ systems that possess a low regenerative capacity. Despite the severity of the problem, HCMV infection of the central nervous system (CNS) remains inadequately characterized to date. Cytomegaloviruses (CMVs) show strict species specificity, limiting the use of HCMV in experimental animals. Infection following intraperitoneal administration of mouse cytomegalovirus (MCMV) into newborn mice efficiently recapitulates many aspects of congenital HCMV infection in CNS. Upon entering the CNS, CMV targets all resident brain cells, consequently leading to the development of widespread histopathology and inflammation. Effector functions from both resident cells and infiltrating immune cells efficiently resolve acute MCMV infection in the CNS. However, host-mediated inflammatory factors can also mediate the development of immunopathologies during CMV infection of the brain. Here, we provide an overview of the cytomegalovirus infection in the brain, local immune response to infection, and mechanisms leading to CNS sequelae.

## 1. Introduction 

Human cytomegalovirus (HCMV), a β-herpesvirus, is a highly prevalent virus infecting 40–100% of the population worldwide [1]. The majority of the infected population remains asymptomatic due to the effective immune response [2]. Upon resolution of primary infection, like other herpesviruses, HCMV establishes lifelong latency. However, primary infection or viral reactivation can cause serious multiorgan disease in immunocompromised individuals. Various risk groups, such as transplant recipients, intensive care patients, acquired immunodeficiency syndrome (AIDS) patients, and fetuses/newborns are susceptible to the development of HCMV-mediated disease due to the impaired immune response [2,3]. In addition, HCMV-mediated life-threatening complications, although rare, are also possible in immunocompetent individuals [4].

### 1.1. Congenital HCMV Infection

Annually, 0.2–2% of all newborns develop HCMV infection in utero, making it the most common congenital infection in the developed world [3,5]. Amongst the infected newborns, 10–15% exhibit clinical findings (symptomatic congenital HCMV infection) such as visceral organomegaly, microcephaly with intracranial calcifications, chorioretinitis, jaundice, mental retardation, sensorineural hearing loss (SNHL), and skin lesions (petechiae and purpura). Additionally, symptoms such as prematurity, small size for gestational age and neonatal death (5–10%) are also considered to define symptomatic congenital infection [1,3,6]. In general, congenital HCMV infection affects more children than widely known trisomy 21 or fetal alcohol syndrome and is a leading cause of non-familial hearing loss [7]. The majority of infected newborns lack clinically evident symptoms (asymptomatic congenital HCMV infection); however, they still possess a high risk of developing neurodevelopmental sequelae, such as SNHL or end-organ disease [3,5]. Studies have reported that even infants that lack evident symptoms can shed virus in their body fluids up to 5 years following the infection, rendering them as a source of virus spread within group care facilities and households [8].

Maternal adaptive immunity can significantly reduce rates of intrauterine transmission, as evidenced by the difference in transmission rates between women undergoing primary infection and women undergoing nonprimary infection [9]. As the severe disease is not observed in the majority of cases, it is evident that most infants can resolve the acute phase of infection without permanent consequences. Observational studies have failed to report an evident connection between viral load in amniotic fluid and the development of long-term sequelae, making estimates of the prognosis of individual cases challenging [6]. At birth, high viral loads in urine and peripheral blood have been correlated with a higher risk of developing sequelae [8,10]. In severe cases, high viral loads can damage fetal organ systems such as hepatobiliary, nervous, hematopoietic, and respiratory [11]. The inability to efficiently resolve acute infections in organ systems that possess low regenerative capacity, such as the nervous and auditory system, can consequently lead to the development of permanent sequelae [11]. It is suggested that the development of severe sequelae is most closely associated with infections during the first trimester in primary infections [12,13]. This observation could be correlated with the undeveloped fetal immune system [14]. The transmission of HCMV to fetus seems to be more common in the last two trimesters [12].

Despite the long history of development, there is no approved vaccine for HCMV [15]. Diagnostic and therapeutic approaches for congenital HCMV infection are still very limited, warranting the need for understanding the pathogenesis of infection and development of novel diagnostic and therapeutic approaches [1]. Usage of antivirals as therapy during pregnancy remains controversial. In theory, antiviral therapy could be effective in preventing fetal infection and in modifying disease in the infected fetuses. However, the majority of antivirals are not approved for use during pregnancy due to their targeting of enzymes required for DNA synthesis, thus leading to possible adverse effects, of which, some could be long-term [12]. Novel antivirals, including letermovir, which is specific for viral terminase, and valacyclovir, are currently being evaluated [12]. The standard postnatal treatment for congenital HCMV infection is the use of antiviral agents such as ganciclovir, which have been shown to control the severity of the acute infection and possibly modify the progression of neurological abnormalities, primarily SHNL [6]. Hyperimmunoglobulin treatment was also tested in clinical trials on pregnant women with confirmed congenital HCMV infection; however, to date, clinical trials have shown no clear benefit of such treatment [16,17]. 

### 1.2. Mouse Model of Congenital HCMV Infection

Cytomegaloviruses (CMVs) show strict species specificity and therefore HCMV pathogenesis cannot be studied in experimental animals. Due to comparable genetics and pathogenesis, various animal CMVs have been used to model HCMV infection [18,19,20]. The most commonly used animal model is the mouse model, but rat, guinea pig, and Rhesus macaque are also frequently used to study CMV infection. 

The Rhesus macaque CMV (RhCMV) model is especially well suited to study congenital HCMV infection in humans [21]. However, this model has major disadvantages, including the paucity of RhCMV-seronegative macaques and the high cost of laboratory animals and experimental setups. The guinea pig CMV (GPCMV) is able to cross the placenta, infect the embryo, and cause pathology in the nervous system [22,23]. However, this model requires high doses of the virus for infection of dams, with consequent significant placental damage, fetal loss, and small litters. In addition, both GPCMV and RhCMV viral genomes lack detailed characterization with an additional shortage of available immunological and genetic tools in comparison to the murine models [22]. Similar drawbacks also apply to rat CMV models of congenital infection [24].

Mouse cytomegalovirus (MCMV) infection has been used to elucidate numerous mechanisms of infection, pathogenesis, and immune response to CMV [25]. Even though MCMV is unable to pass the placenta and infect the embryo, various inoculation techniques have been established to model congenital infection [26,27,28]. Direct inoculation of MCMV into cerebral hemispheres or lateral ventricles of either mouse embryos or newborn mice has been used to model congenital infection [29,30]. However, direct intracranial (i.c.) inoculation of the virus does not efficiently reflect the pathogenesis of congenital infection, including a disregard of viral spread and immune response in peripheral tissues prior to infection of the CNS. Additionally, these methods require pretreatment anesthesia and complex techniques and can lead to significant loss of animals in experimental groups in comparison to intraperitoneal (i.p.) inoculation, as it can lead to collateral infections and disruptions of the blood–brain barrier (BBB) [26,31]. Intraperitoneal inoculation of MCMV into newborn pups is a commonly used method for studying congenital infection in mice [26]. The use of newborn mice is justified by the fact that the CNS of neonatal mice corresponds developmentally to the CNS of human fetuses between gestation weeks 12 to 15 and by the highly conserved structure of the cerebellum between rodents and humans [31,32,33]. Following inoculation, the virus spreads hematogenously, establishing primary viremia in peripheral organs prior to infecting the CNS, which resembles the proposed route of HCMV dissemination into CNS during congenital HCMV infection [26,31]. Importantly, infected mice develop brain alterations and neurobehavioral sequelae observed in congenital HCMV infection [26,34]. Similarly, MCMV-infected newborn mice exhibit hearing loss associated with loss of spiral ganglia neurons and degeneration of cochlear vasculature [35,36]. Newborn mice of different strains show different levels of susceptibility to MCMV infection [37,38], potentially resembling differential susceptibility for symptomatic infection in the human population. However, only BALB/c and C57BL/6 mice have been used to model congenital infection so far. The kinetics of virus replication and virus-induced pathology are similar in newborn BALB/c and C57BL/6 mice [39,40,41]. Ly49H receptor, which provides MCMV resistance in C57BL/6 mice, is not expressed in NK cells in newborn mice, explaining the lack of efficient control of MCMV in newborn C57BL/6 mice [41,42].

## 2. CMV Infection of Brain-Resident Cells

### 2.1. Cytomegalovirus Tropism

CMV possesses a broad cell tropism, with the majority of cell types reported to be fully permissive for infection [43]. Cell types such as epithelial, endothelial, fibroblasts, and smooth muscle cells are considered to be the prime targets for HCMV infection [44]. It is presumed that HCMV enters a new host by infecting mucosal epithelium, or in the case of congenital infection, by infecting placental trophoblasts. Upon entry, HCMV establishes myeloid cell-mediated primary infection of organs such as spleen, liver, and lungs [45]. Efficient proliferation in ubiquitous cell types such as fibroblasts, hepatocytes, and smooth muscle cells contributes to high viral loads in different organs [44]. Interestingly, even though the liver is one of the organs with the highest viral load during acute infection in adult mice, the hepatocyte-produced cytomegalovirus does not disseminate [46]. Myeloid cells are considered to function as transport vehicles for viral spread, rather than as sites of robust productive infection. Following infection of initial organs, the virus undergoes secondary dissemination to organs such as salivary glands and kidneys [45]. Infection of epithelial cells of glands and mucosal tissues allows the virus to spread to new hosts via infected bodily secretions. 

Three major glycoprotein complexes of cytomegalovirus, gB, gM/gN, and gH/gL, mediate virus entry [47]. Their contributions to entry are thought to occur sequentially, with the gM/gN complex mediating initial attachment to the host cells, gH/gL complexes binding cell surface receptors, and gB mediating membrane fusion [48]. The binding of gH/gL complexes to an entry receptor induces conformational changes that activate gB to perform membrane fusion [48]. HCMV encodes two gH/gL complexes. The trimeric complex consisting of gH/gL and gO (gH/gL/gO) mediates entry into all cells, most notably fibroblasts, and is required for infectivity of cell-free virus [49]. The pentameric complex of gH/gL with small glycoproteins UL128, UL130, and UL131 broadens the HCMV cell tropism, and is required for infection of epithelial cells, endothelial cells, leukocytes, and dendritic cells, but not fibroblasts [50,51,52,53]. The ability of HCMV to efficiently infect various cell types can be also linked to the efficient exploitation of numerous host surface receptors and co-receptors that mediate viral entry [48]. The gH/gL/gO complex mediates infection of fibroblasts by binding to platelet-derived growth factor receptor-α (PDGFRα), a receptor that is not expressed on epithelial cells [54,55]. The pentameric complex targets neuropilin-2 (Nrp2) for efficient infection of epithelial and endothelial cells [56].

Similar to HCMV, MCMV shows broad tropism and encodes two gH/gL complexes [47]. Mouse gH/gL/gO complex is a functional homolog of the corresponding HCMV complex and it is important for infectivity of MCMV virions and fibroblast tropism [57]. The second complex of MCMV gH/gL is with viral chemokine-like protein, MCK-2 [58]. gH/gL/MCK-2 mediates cell-associated spread, infection of macrophages, and dissemination to salivary glands [58,59]. Interestingly, while gH/gL/gO is critical for establishing infection, both gH/gL/MCK-2 and gH/gL/gO can mediate intra-tissue spread [60]. The role of CMV glycoprotein complexes in infection of the CNS, spread, and neurotropism is yet to be established. 

### 2.2. Cytomegalovirus Infection of Neurons and Glial Cells

The viral transmission from HCMV-positive mothers to fetuses starts at the uterine–placental junction by first infecting uterine smooth muscles and endothelial cells in the decidua [61]. Secondly, virus interactions with trophoblast cell receptors mediate transplacental transmission, which consequently enables the virus to enter the fetal blood system [61,62]. It is suggested that the virus enters the fetal circulation in a cell-free form due to the placenta’s limited permeability for maternal cells to enter fetal circulation [3]. When HCMV crosses the placenta into the fetal blood system it undergoes replication in numerous fetal organs [63]. However, the exact dissemination pathway from the maternal placenta to individual organs, including the brain, is still not resolved [3]. 

**Figure 1:**
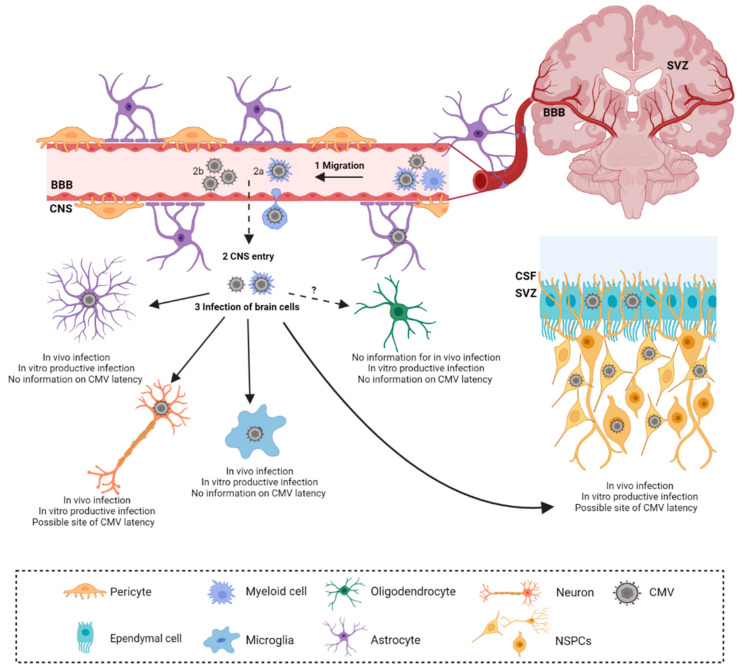
Cytomegalovirus infection in developing brain. Cytomegalovirus (CMV) dissemination to the central nervous system (CNS) is secondary to peripheral organ infection (1). Upon reaching the brain, CMV is hypothesized to cross the blood–brain barrier (BBB) by either cell-associated (2a) or cell-free form (2b). Monocytes are proposed to mediate cell-associated passage across the BBB. Upon crossing of the BBB, CMV infects resident cells (3). Apart from oligodendrocytes, CMV infection of resident CNS cells was confirmed in vivo. CMV DNA was detected in cerebrospinal fluid (CSF) of congenitally infected infants and neural stem precursor cells (NSPCs), abundant in subventricular zones (SVZ), are a prominent target of CMV infection. Figure was created with Biorender.

Studies of HCMV infection in the brain during congenital infection are limited mainly to histopathological and observational studies. Additionally, discrepancies in the timing of fetal infection, viral burden, and wide variations in histopathological changes, accompanied by a lack of non-invasive methods, impair direct study of HCMV in the brain [25]. Therefore, the mouse model has been informative in defining the kinetics of virus spread and subsequent immune response and pathology. It has also been suggested that the neonatal brain is more susceptible to MCMV infection following i.c. infection as compared to the adult brain [64]. Furthermore, peripherally infected adult mice are resistant to the invasion of CMV into the brain, due to efficient immune control [65]. As the virus can be detected in both the plasma and blood upon i.p. inoculation of MCMV into newborn mice, it is assumed that it can enter the CNS in both forms, cell-free or cell-associated; however, the exact mechanism of crossing the blood–brain barrier is still unknown [66]. Mononuclear cells are speculated to be the entrance gates for the viral migration into the developing CNS [26,31,65]. Following i.p. infection, the infectious virus can be isolated from the brain starting from 7 days post-infection (d.p.i.) up to 21 d.p.i [39,40]. The virus efficiently infects all cell types in the brain, showing no specific cell tropism in the CNS (Figure 1) [67]. However, the vast majority of the published data are based on in vitro analysis from primary cell cultures [67]. Additionally, discordant findings have been reported by different research groups in the capacity of resident CNS cells to support a full viral replication cycle. 

Astrocytes are the most abundant glial cells with a range of diverse functions ranging from metabolic support to regulation of synaptogenesis [68]. As astrocyte foot processes are involved in the formation and maintenance of the blood–brain barrier, they are therefore resident cells of the CNS that are the initial targets of neurotropic viral infections [69,70]. During acute infection, histopathological studies of fetal brains have shown that among resident cells, GFAP^+^ astrocytes represent the predominant cell type infected with HCMV (Table 1.) [71]. In vitro, astrocytes are fully permissive for HCMV and MCMV and support productive replication [72,73,74,75,76]. 

Microglia are yolk sac-derived, tissue-resident immune cells of the CNS [80]. During development, microglia populate the CNS and establish a long-lived cell pool [81]. This cell type possesses an abundance of specific proteins referred to as the sensome, that enable microglia to efficiently respond to neurotropic viruses and other microbes that invade the CNS, rendering them essential in protection against viral encephalitis [82]. Early in vitro analyses of HCMV infection of enriched microglia cell cultures reported opposing results on HCMV’s ability to infect this cell type (Table 1) [72,74,83,84]. By analyzing fetal brains, separate research groups have confirmed that microglia are indeed susceptible to CMV infection during acute congenital HCMV infection, embryogenesis in RCMV, and MCMV infection of newborn mice [41,71,85]. In the case of HCMV infection, microglia are not a primary target of HCMV, accounting for approximately 10% of infected cells [71]. In vitro, microglia were reported to be permissive for MCMV infection and to support productive viral replication [86]. Furthermore, MCMV productively infects both ramified/quiescent and amoeboid/activated phenotypes of the BV-2 microglial cell line [87]. 

Neurons possess a unique cell morphology which enables them to perform vital functions of receiving and sending electrical impulses [88]. One of the particular features of neurons is limited regeneration; once fully differentiated, neurons possess poor regeneration capabilities upon damage [89]. Therefore, neurons have developed pro-survival strategies to avoid destruction from cellular components of the immune system [90]. This self-preservation evolutionary feature is often exploited by neurotropic viruses in both the CNS and peripheral nervous system (PNS) [90]. Additionally, it is postulated that viruses exploit neuronal metabolism by hitchhiking with the axonal traffic [90]. Histopathological examination of brains from fetuses with severe manifestations of intrauterine HCMV infection reported on neuron infection, accompanied by increased levels of apoptosis (Table 1) [63,71,91]. However, neurons are reported to be infected to a lesser extent than resident glia cells, as only a few post-mitotic HCMV-positive neurons were observed [71]. In vitro, conflicting results have been reported on the permissiveness of HCMV infection in neurons, ranging from no infection to full permissiveness [72,92,93,94]. Interestingly, neural stem precursor cell (NSPC) differentiated neurons were associated with a decrease in viral replication in comparison to their undifferentiated precursors [93]. This phenomenon is hypothesized to be correlated with either efficient control of IE major promoter (MIEP) activity or viral exploitation of the poor neuronal antiviral response [93,95]. The expression of the MIEP is thought to be regulated by various transcription factors that respond to cell differentiation or membrane polarization, while the latter can be correlated with low expression of MHC molecules on neurons, a phenotype that is believed to protect neurons from immune-mediated cell destruction [95,96]. MCMV infection of neurons has been reported in the developing brain following both peripheral and cranial inoculation models [34,64,97]. 

During embryonic development of the CNS, both neurons and glial cells (except microglia) develop from NSPC, a common neuroepithelial precursor [98]. A population of NSPCs is present in adulthood as well and resides in subgranular zones of the dentate gyrus and ventricular zones of the cerebellar cortex [99]. Histopathological analysis of brains from infants with severe congenital HCMV infection revealed a high number of infected cytomegalic cells, and loss of germinal and radial glial cells in proximity of ventricular zones (Table 1) [100]. These observations suggest that NSPCs could be a prime target for HCMV infection. Primary NSPCs isolated from human fetuses or neonates have been shown to be fully permissive and support active viral replication with both early and late gene expression [92,93,101,102]. Similar data were reported for in vitro MCMV-infected mouse NSPCs, which support productive viral replication [103,104]. Interestingly, neural stem cells are more resistant to HCMV infection in comparison to neural progenitor cells, which have limited proliferative ability and do not exhibit self-renewal [105]. Histopathological brain analysis of severe cases of intrauterine HCMV infections established that the virus favors ventricular regions which have abundant NSPCs [100]. Teissier et al. reported that during intrauterine HCMV infection, NSPCs are indeed the prime targets of CMV infection, as the majority of HCMV-positive cells show hallmarks of NSPCs [71]. Additionally, similar extensive infection of NSPCs was reported in the murine i.c. model of infection [77,104,106].

Oligodendrocytes are glial cells that are predominantly located in white matter and are responsible for the myelination of axonal membranes [107]. Infection of oligodendrocytes with CMV is poorly characterized [78]. The human oligodendroglioma cell line (HOG), which mimics immature oligodendrocytes, is permissive for HCMV infection, and productive infection was observed exclusively in stimulated HOG cells which resembled mature oligodendrocytes (Table 1) [78]. 

Ependymal cells are cuboidal, glial cells that form a single layer around the ventricular system of the brain and the central canal of the spinal cord. It is suggested that ependymal cells provide trophic and metabolic support. Due to the direct contact with cerebrospinal fluid (CSF), it is not unexpected that ependymal cells are targets for viral infection [108]. Histopathological examinations of congenitally infected fetuses confirmed HCMV infection of ependymal cells (Table 1) [79,100]. In addition, primary ependymal cultures were also permissive for HCMV infection [109]. Ependymal cells lining the ventricles have been reported to be highly susceptible to infection in the MCMV i.c. model [64].

### 2.3. Cytomegalovirus Latency in Brain

One of the signatures of the herpesvirus family is the establishment of a life-long latency from which the virus intermittently reactivates [110]. By limiting viral replication during latency, CMV efficiently avoids host immune cell activation, while maintaining the viral genome in host cells. Even though CMV latency is extensively studied, this viral state is still not well understood [111,112,113]. In contrast to herpes simplex virus 1 (HSV-1), where latency-associated transcripts (LATs) are well characterized, genes expressed during CMV latency are not latency-specific, as their expression was observed also during the lytic cycle [112,114,115]. Consistent with these observations, recent studies indicate that a hallmark of HCMV latent infection is a low-level expression of a broad spectrum of canonical viral lytic genes [116,117,118].

In primitive neural stem cell (pNSC) culture, HCMV genomes were detectable up to one month after infection, without any detectable IE1 expression, suggesting NSPCs as a reservoir of latent HCMV (Table 1) [119]. In contrast, by using fetal-derived NSPCs it was suggested that neurons act as a reservoir of latent HCMV [92]. The human embryonal carcinoma cell line NTera2, which can be differentiated into neurons upon retinoic acid treatment, was used to determine molecular mechanisms of latency in neuronal progenitor cells [111,113]. It was observed that stimulation of the cAMP signaling pathway activates viral reactivation in NTera2 cells, suggesting a possible role in CMV latency [120,121]. 

MCMV can be reactivated from latency in brain slice cultures of mice infected as newborns [122]. A high degree of infection was observed around ventricular zones following reactivation, suggesting that NSPCs could function as a viral reservoir during latency. Furthermore, upon loss of immune control MCMV reactivates in the brain in vivo in mice infected as newborns [40,123]. Depletion of either CD8^+^ or CD4^+^ T lymphocytes was sufficient to achieve reactivation; however, the identity of cells reactivating MCMV in this context remained undetermined [40,123]. 

The impact of latent CMV infection on the homeostasis and function of the nervous tissue is currently unclear. It was demonstrated that latent MCMV infection in the brain promotes the development of glioma [124]. This is in line with the hypothesis that HCMV could be an oncomodulatory agent in developing gliomas [125]. Therefore, latent CMV in CNS could be involved in the development of a range of diseases.

## 3. Immune Response to Cytomegalovirus Infection in Developing Brain 

**Figure 2:**
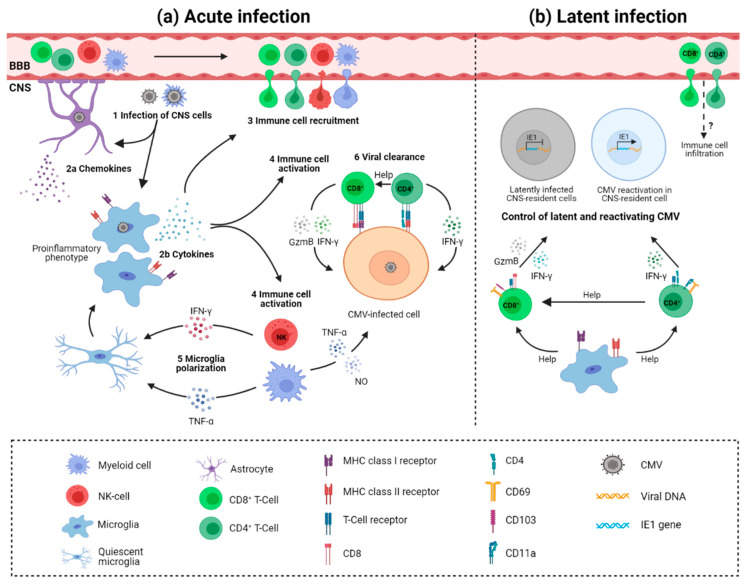
Immune response to cytomegalovirus infection in the brain. (**a**) Acute infection. Upon crossing of the blood–brain barrier (BBB), cytomegalovirus (CMV) infects resident cells (1). Astrocyte-derived chemokines recruit microglia to the infection site (2a). Microglia are activated via pattern recognition receptors and cytokines. Activated microglia produce proinflammatory cytokines (2b), which mediate immune cell recruitment into the brain (3) and orchestrate immune cell response (4). Infiltrating NK cells and ILC1 cells produce IFN-γ and myeloid cells produce TNF-α, leading to organ-wide polarization of microglia (5); infiltrating T cells provide direct control of productive infection (6). CD8^+^ and CD4^+^ T cells recognize virus-infected cells in the context of MHC I and MHC II molecules and provide virus control by cytolytic mechanisms (gzmB) or by non-cytolytic mechanisms (IFN-γ). (**b**) Latent infection. Following resolution of acute CMV infection, T cells are retained in the brain as tissue-resident cells (T_RM_) and control latent/reactivating CMV. CD8^+^ T_RM_ cells are characterized by expression of CD69 and integrin CD103, while CD4^+^ T_RM_ cells express CD69 and CD11a. Both cytolytic mechanisms (gzmB) and cytokines (IFN-γ) could mediate the control of latent and reactivating CMV in the CNS. T_RM_ cells are suggested to persist in the brain of mice for a lifetime without or with minimal replenishment from the circulation. Activated microglia probably contribute to maintenance and functional capacity of TRM cells in the brain. Figure was created with Biorender.

Upon cytomegalovirus infection of the brain, different resident and infiltrating cells mediate protection (Figure 2) [126]. Astrocytes are probably the first brain cells exposed to infection due to their location surrounding blood vessels. So far, there is no definitive evidence of astrocyte-mediated control of CMV infection. However, as seen in other infections, astrocytes could have an important role in CNS innate immunity as they express various pattern recognition receptors (PRRs). Activation of PRRs such as Toll-like receptors (TLR) leads to downstream expression of interferon-stimulated genes, consequently establishing an antiviral immune response [127]. Supernatants of HCMV-infected primary astrocyte cultures contain high levels of chemokines that could attract microglia to the infection site [84]. The migration of microglia to the infection foci is probably a source of proinflammatory cytokines and mediators of antiviral response [84]. By using i.p. MCMV infection of newborn mice we have shown that microglia acquire a proinflammatory phenotype and transcriptional profile, proliferate, and produce antiviral cytokines during acute infection [41]. While the direct role of microglia in the control of MCMV infection has not been shown, microglia likely have a major role in orchestrating immune response in the brain. Besides microglia, CNS-associated macrophages are activated and peripheral blood monocytes infiltrate the brain early after infection [26,39,128,129]. Activated microglia are not limited to the viral foci, but are rather equally distributed, favoring the hypothesis that virus infection of the developing brain results in a widespread pro-inflammatory response [129]. Expression of genes involved in interferon response (IRF-1, IRF-7, USP18, LRG-47, IFIT1, STAT1), pro-inflammatory cytokines (TNFα, IFNβ, IL-1β, IFNγ), chemokines (CXCL10, CCL2, CCL5, CCL21), and both MHC class I and MHC class II molecules are shown to be significantly elevated and widely expressed in the cerebellum of infected animals [26,128,129]. NK and ILC1 cells infiltrate the brain as well and produce IFN-γ (Figure 2a). This early IFN-γ production leads to polarization of microglia; however, it does not contribute to virus control in the brain [41]. Recent studies suggested that neurons are actively involved in the CNS immune response [96]. However, the involvement of neurons in immune responses to CMV infection is yet to be determined.

The importance of adaptive immunity, especially T cells, in controlling CMV infection is well established in immunocompromised individuals and murine models [130]. Both CD4^+^ and CD8^+^ T cells infiltrate the brain following the infection of newborn mice [39]. Virus replication in the CNS is shown to resolve gradually following the increasing levels of infiltrating CD8^+^ T cells (Figure 2a) [39,40]. Depletion of CD8^+^ T cells results in a significant increase in viral load in the brain and peripheral organs and mortality [39]. Even though the levels of CD4^+^ T cells are much lower as compared to CD8^+^ T cells in the brain of infected mice, they are similarly important for the control of virus replication in the brain and resolution of productive infection [123]. It is well established that maternal antibodies reduce the risk of HCMV transmission to the fetus, as well as improve disease outcome [131]. Similarly, we have shown that offspring of MCMV-immunized mothers are protected from MCMV infection [132,133]. In addition, adoptive transfer of immune sera or monoclonal antibodies specific for viral glycoproteins can reduce MCMV levels in newborn mice, as well as the development of pathology [134].

The control of the latent virus in the brain is less well understood. Following resolution of MCMV infection, T cells persist in the brain of mice for the lifetime of the animal [40]. Persisting T lymphocytes are characterized by the establishment of a tissue-resident memory phenotype (T_RM_), as CD8^+^ T cells express CD69 and CD103, and CD4^+^ T cells express CD11a and CD69. In addition, CD8^+^ T_RM_ cells express elevated levels of PD1, CD44, TCR, and co-receptor CD8, and are long-lived slowly proliferating cells [40]. Phenotypic and functional analysis of CD4^+^ T_RM_ cells has shown that they express Th1 markers (T-bet and CXCR3) and cytokine IFN-γ [40,123]. Importantly, T_RM_ populations are functionally competent and provide protection upon reinfection (Figure 2b) [40]. Long-term depletion of either CD4^+^ or CD8^+^ T cells from latently infected brains results in the appearance of MCMV IE1^+^ cells in the brain [40,123]. Interestingly, depletion of CD4^+^ T cells from the brain results in the loss of T_RM_ marker CD103 expression by CD8^+^ T cells. Whether loss of CD103^+^ population of CD8^+^ T cells results in impaired control of latent virus remains undetermined. The importance of CD8^+^ T cells is not limited to control of virus reactivation, but they also provide control of the inflammatory response in latently infected CNS [40].

### Pathogenesis of Congenital CMV Infection in the Brain

Congenital HCMV infection-induced neuropathology in the CNS is widespread. Lesions are found in different regions of the brain such as the hippocampus, olfactory bulb, eyes, and inner ears, which leads to impaired perceptual senses (SNHL, chorioretinitis) or neurological diseases accompanied with structural deformity [12,100]. Histopathological changes are manifested in the form of cerebellar and cortical hypoplasia (underdevelopment or incomplete development of the brain), microcephaly (reduction in head size), meningoencephalomyelitis (inflammation of the meninges, brain, and spinal cord), neuronal heterotopia (atrophy of the cortical plate and rupture of the glia limitans), ventriculomegaly (larger ventricles than normal), calcifications in the form of nodules, hemorrhagic lesions, hemosiderosis (iron overload disorder), necrosis, and cellular loss [100]. Due to the limitations of observational studies, the exact mechanism of pathogenesis remains unresolved. It is suggested that it involves disruption in the microvasculature of the developing brain, damaged blood–brain barrier, altered synaptogenesis, loss of NSPCs, and altered cell migration manifested in disordered cellular positioning [26,66,100,134]. 

The MCMV model of congenital infection efficiently recapitulates many aspects of the neuropathology associated with congenital HCMV infection [26]. Namely, focal and non-necrotizing encephalitis are observed in brains of i.p.-infected newborn mice, accompanied by mononuclear cell infiltrates and alterations in cerebellar morphology and size [26]. No striking differences were observed in the cerebrum of infected animals [26,134]. However, the cerebellum is part of the brain that undergoes extensive postnatal development as opposed to the cerebrum, making it highly susceptible to viral-mediated perturbation [135]. The observed cerebellar pathology parallels the viral kinetics in the CNS. Upon resolution of acute viral infection, the cerebellar growth is normalized, suggesting virus-mediated growth retardation [26,39,40]. Importantly, similar morphological changes in cerebellum size are observed in acute cases of congenital HMCV infection [136]. Additionally, global histopathological lesions such as edema, micronodular gliosis, perivascular cuffing, and reactive gliosis are also observed in the brains of infected newborns and can persist to a lower extent following resolution of acute infection [134].

Alterations in NSPC differentiation and migration can lead to extensive malformations in cortical development and manifest as severe pathology [137]. NSPCs have a reduced ability to proliferate and differentiate into neuronal and astrocyte lineage during productive HCMV infection as shown in vitro [101,102]. It is proposed that these alterations in differentiation correlate with IE1-specific targeting of STAT3 phosphorylation, which consequently decreases levels of SOX2 expression, a transcription factor (TF) crucial in NSPCs pluripotency and self-renewal [138]. Besides the STAT3–SOX2 pathway, IE1 is shown to function as E3 ubiquitin ligase, which targets and downregulates Hes1, a TF involved in downstream Notch signaling, essential in NSPC differentiation and brain development [139]. The ability of CMV to induce NSPC apoptosis is still unclear as conflicting findings have been reported in studies to define the role of HCMV in NSPC apoptosis in vitro [93,102]. However, histopathological examination of HCMV-infected brains reported extensive cell loss and necrosis in brain zones abundant with NSPCs [100]. 

Similar data were reported for in vitro MCMV-infected mouse NSPCs that support productive viral replication and have reduced ability to proliferate and differentiate. Additionally, alternations in cellular processes such as DNA synthesis, self-renewal, migration, and downregulation of MHC class I molecules were also observed [103,104]. These data were validated by i.c. MCMV infection of newborn mice, resulting in infection of NSPCs and causing a substantial decrease in NSPC number, proliferation, and self-renewal while also disrupting their differentiation into a neuronal lineage. Disruption in neurogenesis was linked with decreased expression levels of neurotrophins such as brain-derived neurotrophic factor (BNDF) and neurotrophin-3 (NT3) [77]. Whole-genome expression analysis on cultured human NSPCs infected with HCMV also reported alterations in gene expression and mRNA levels of genes important for NSPC differentiation. The authors suggested that this change in gene expression likely correlates with premature and abnormal differentiation [140]. 

In MCMV-infected newborn mice, increased thickness of the cerebellar external granular layer (EGL) and decreased thickness of the internal granular layer (IGL) and molecular layer (ML) can be observed [26,128,129,134]. The thicker EGL is correlated with an increase in cellularity of granule neuron precursor cells (GNP), while decreased thickness of the IGL is hypothesized to develop secondary, as a result of reduced granular neuron migration [26,128,129]. The Purkinje cell body size did not differ between the infected and control group. However, lower cell numbers of Purkinje cells were observed, accompanied by impaired alignment and decreased dendrite arborization, consequently leading to a decreased thickness in the ML [26,134]. It was suggested that GNP cells have lower proliferation and migration rates, while increased levels of apoptosis were not observed [26]. Further studies indicated the increased ratio of GNP cells in the S phase without a decrease in the number of cycling cells [128,129]. This observation would suggest that CMV infection blocks or delays GNP proliferation downstream from gli1 and N-myc, effectors of granular neuron proliferation in the Sonic hedgehog (SHH) pathway that are elevated during MCMV infection [128,129,141]. Prolongation in the GNP cell cycle would delay the expression of developmental genes that are directly connected to adequate positioning and differentiation of GNPs, consequently leading to a reduction in cerebellar foliation and cerebellar size, and altered EGL thickness. Additionally, MCMV infection decreased activation of neurotrophin receptors, which are actively involved in postnatal cerebellar development [26,142]. Furthermore, differentiation of GNP cells has also been shown to be altered with an observed reduction in the expression of molecular markers for granular neuron differentiation and differentiated neurons [26,128,129]. The reported data are somewhat similar to the reported data on NSPC infection in vitro as CMV infection efficiently alters normal GNP processes such as proliferation and differentiation.

The involvement of other CNS cell types in altered neurodevelopment is poorly studied. Analysis of MCMV-infected neurons reported impaired homeostatic processes, such as neuronal conductivity, attenuation in generating action potentials, and synaptic activity, while maintaining normal morphology during MCMV infection [73,143]. MCMV infection of primary astrocytes alters intercellular communication in vitro [73]. The observed increase in levels of intracellular calcium (Ca^2+^) in MCMV-infected astrocytes consequently diminished neuronal synaptic activity and intercellular communication between astrocytes [73]. Interestingly, perturbations in Ca^2+^ signaling were reported to alter neurogenesis in ventricular zones, while also increasing susceptibility for neuron infection in the HSV-1 model [144]. These data suggest a possible connection between CMV-mediated alteration in intercellular communication and consequently diminished neurogenesis. Whether oligodendrocytes are involved in altered neurodevelopment during congenital HCMV infection is not known. In humans, myelination begins around gestation week 30, and continues extensively in the postnatal period and during the first year, and therefore it is possible that oligodendrocytes and myelination are not significantly affected by HCMV infection prior to birth [145]. 

While the protective role of the host inflammatory response is clear, there is a delicate balance between neuroprotection and neuropathology [126,146]. Infection can cause excessive activation of astrocytes and microglia, consequently overproducing proinflammatory mediators. This immune imbalance can lead to oxidative stress, tissue degeneration, neuronal death, and cognitive decline [146]. Additionally, an excessive immune response can mediate various neurodevelopmental disorders [147]. Therefore, one can argue that host immune response to CMV infection in the brain is neuropathologic, as observed morphological alterations are not limited solely to adjacent foci of infection or to immune cell infiltrations but are rather more globally distributed. This could suggest that the observed morphological changes are not directly correlated with viral cytopathic activity, but rather with the host pro-inflammatory response [31,66]. Indeed, we have previously shown that glucocorticoid treatment of MCMV-infected newborn mice attenuates CNS inflammation and limits deficits in cerebellar development, while minimally affecting virus replication [128]. Such treatment limited morphogenic abnormalities, normalized the expression of developmentally regulated genes within the cerebellum, and normalized GNPC proliferation deficits. Further studies indicated that TNF-α is a major component of the inflammatory response associated with altered neurodevelopment in the MCMV-infected developing brain, with key effector cells in this process being myeloid cells [128]. NK/ILC1 cell-derived IFN-γ similarly exerts a detrimental impact on cerebellar development in the infected developing brain [41]. Conversely, blocking of TNF-α or IFN-γ, or depletion of NK cells, normalizes cerebellar development. Altogether, these studies demonstrate that limiting the proinflammatory response can alleviate the CMV-induced pathology and open the potential therapeutic avenues. Similar mechanisms could be responsible for CMV induced hearing loss, as decreasing cochlear inflammation by corticosteroid treatment of MCMV-infected mice resulted in preservation of spiral ganglion neurons and improved auditory function [35]. Whether other immune cells play a detrimental role in altered neurodevelopment in MCMV-infected developing brain is not known. Furthermore, the sequence of events leading to altered neurodevelopment, as well as interactions of inflammatory mediators and cells, are currently unknown.

## 4. Closing Remarks and Future Perspectives

Many important aspects of congenital HCMV infection in the CNS, such as mechanisms of viral entry and dissemination, induction of immune response, and development of pathologies, remain ill-defined and incompletely understood. The mouse model of congenital HCMV infection provides an opportunity to define some of these mechanisms. While it is clear that CMVs can infect (almost) all brain cell types, the mechanisms which mediate virus dissemination to the CNS, the spread of the virus in the CNS, and the consequences of infection of individual cell types, are still unknown. Major viral glycoprotein complexes, and especially gH/gL complexes, could be essential in guiding the virus to the CNS and are considered to be an important target of neutralizing antibodies. Therefore, future studies should address their involvement in CMV infection of CNS, as well as the potential of their blocking. Host immune response can be regarded as a double-edged sword; besides providing virus control, neurodevelopmental pathology can be corrected by suppressing the immune response. Defining mechanisms of immunity mediating these detrimental outcomes could potentially provide hints for the development of interventional therapies. Once the acute infection is resolved in the CNS by an efficient host immune response, primarily CD4^+^ and CD8^+^ T cells, the virus establishes latency, from which it can reactivate upon loss of immune control. However, the impact of the latent virus on the homeostasis and function of this delicate tissue is currently unknown. The lifelong persistence of T cells in the CNS, which provide control of the latent virus, warrants the need for future studies to define their role in the development of different pathological conditions. 

## Figures and Tables

**Table 1:** HCMV and MCMV infection of brain-resident cells.

Cell Type	Acute Infection		Latent Infection	Selected References
	In Vivo	In Vitro		
Astrocytes	HCMV (+) MCMV (?)	HCMV (+) MCMV (+)	No information	[71] [72]
Microglia	HCMV (+) MCMV (+)	HCMV (+/−) MCMV (+)	No information	[71] [41]
Neurons	HCMV (+) MCMV (+)	HCMV (+/−) MCMV (?)	Possible site of latency	[71] [34]
NSPCs	HCMV (+) MCMV (+)	HCMV (+) MCMV (+)	Possible site of latency	[71] [77]
Oligodendrocytes	HCMV (?) MCMV (?)	HCMV (+) MCMV (?)	No information	[78]
Ependymal cells	HCMV (+) MCMV (+)	HCMV (+) MCMV (?)	No information	[79] [64]

Human cytomegalovirus (HCMV), murine cytomegalovirus (MCMV), neural stem precursor cell (NSPC), infected (+), not infected (−), no information (?).

## Data Availability

Not applicable.
